# Comprehensive *in silico* analysis of genetic landscape and pathways involved in Stickler syndrome

**DOI:** 10.1371/journal.pone.0343405

**Published:** 2026-02-20

**Authors:** Ravinder Sharma, Kiran Yadav, Vikas Gupta, Anchal Arora, Vikas Yadav

**Affiliations:** 1 Faculty of Pharmaceutical Sciences, The ICFAI University, Himachal Pradesh, India; 2 University Centre of Excellence in Research, Baba Farid University of Health Sciences, Faridkot, Punjab, India; 3 Department of Pharmaceutical Sciences and Drug Research, Punjabi University, Patiala, Punjab, India; 4 Department of Clinical Sciences, Clinical Research Centre, Skåne University Hospital, Lund University, Malmö, Sweden; Shaheed Rajaei Cardiovascular Medical and Research Center: Rajaie Cardiovascular Medical and Research Center, IRAN, ISLAMIC REPUBLIC OF

## Abstract

Stickler syndrome is a collection of hereditary conditions that impact connective tissue, mainly collagen, and can cause a variety of symptoms, such as joint and bone abnormalities, hearing loss, and visual impairments. Previous studies suggest that mutations in the collagen-encoding genes are a primary cause of SS. These mutations can be inherited from parents to offspring and may vary significantly in terms of severity and symptoms. Besides these mutations, the complex genetic maze underlying SS remains poorly understood, limiting the development of targeted therapeutic and biomarker options. In this study we aimed to identify key genes and molecular pathways potentially involved in SS using bioinformatics approaches, and to explore putative therapeutic directions. In our text mining analysis, we identified 24 distinct genes associated with SS in *Homo sapiens*, out of which 22 were chosen as candidate genes for enrichment analysis, based on their Gene Ontology (GO) annotations and participation in pertinent biological pathways. Cytoscape-based construction of the protein–protein interaction network revealed a single functional module comprising 22 nodes and 46 edges, from which nine hub genes were identified. Enrichment analysis demonstrated that these genes were predominantly involved in extracellular matrix organization, collagen fibril organization, skeletal system development, and extracellular structural organization, all of which play a critical role in the pathogenesis of SS. Furthermore, drug-gene interaction analysis suggested six of the nine hub genes may be linked to FDA-approved compounds. Our results provide a systematic framework for prioritizing genes and pathways which may pave the way for future studies aimed at biomarker discovery and therapeutic exploration in SS.

## Introduction

Stickler et al. originally described Stickler syndrome (SS), a connective tissue condition, in 1965. It is often referred to as progressive hereditary arthro-ophthalmopathy. The frequency of this uncommon hereditary illness ranges from 1/7500–1/9000 births, rendering it as an orphan disease condition [1]. SS is characterized by anomalies of the eyes, hearing loss, joint issues, and a unique facial look. A flattened facial look caused by undeveloped bones in the middle of the face is a hallmark of SS [2]. Patients with SS also frequently exhibit a specific set of physical characteristics known as the Pierre Robin sequence [3]. Many people with SS suffer from extreme myopia, which makes it difficult for them to view distant objects. Sometimes the transparent gel used to fill the eyeball looks strange. SS patients also frequently experience other eye issues, such as elevated intraocular pressure, clouding of the lens, and tearing of the light-sensitive tissue at the rear of the eye. Vision impairment and blindness may result from certain eye disorders [4,5]. People with SS also frequently experience hearing loss. The extent of hearing loss in those who are impacted can vary and may get worse with time [6]. The majority of SS patients have problems in their joints. Arthritis can cause joint pain or stiffness and frequently appears early in life. In their 20s or 30s, those with severe joint disease can require surgery to replace destroyed joints. People who are affected may also have kyphosis or scoliosis, which are abnormal curvatures of the spine that cause back pain [7,8].

SS has a complex genetic makeup as well. Some types are inherited in an autosomal recessive fashion, while the most prevalent types are autosomal dominant. The dominant mutations linked to SS have been found in three distinct genes that encode collagen types 2 (COL2A1) and 11 (COL 11A1 and 11A2, respectively), thanks to recent advancements in molecular genetics [9]. There have also been reports of other uncommon mutations that are passed down in an autosomal recessive manner, such as collagen 9 mutations (COL 9A1, COL 9A2, or COL 9A3) [10]. SS is often classified into three categories based on the mutation and the patient’s ocular characteristics. The most common form (approximately 80%) is linked to a COL2A1 mutation, type 2 (approximately 20%) to a COL11A1 mutation, and type 3 (approximately 1%), to a COL11A2 mutation [11]. There aren’t many statistically evaluated studies on these manifestations, and it’s unclear how common they are. They are believed to be connected to the crucial role collagen plays in postnatal growth and the formation of the embryonic cartilage skeleton. In rare cases, it has been discovered that people who exhibit symptoms and indicators resembling those of SS have genetic variations unrelated to collagen synthesis. Finding trustworthy biomarkers and novel medications are required for SS patients, since both have not yet been determined and established in clinics. Integrative approaches that combine several data sources, analytical tools and text mining have gained popularity recently among researchers looking to uncover genes in rare or complex illnesses [12]. Additionally, network modelling of gene-gene and/or protein-protein interactions provides fresh perspectives for comprehending and discovering components linked to disease. By using text mining of biomedical literature and combining it with the Drug-Gene Interaction database, we attempted to explore the genetic landscape of SS and find a putative therapeutic direction for the same. While our *in silico*-based findings may provide a useful hypothesis and a foundation for future investigations, their biological and clinical relevance warrants further validation through experimental and clinical studies.

## Methods

### Text mining for genes associated with stickler syndrome

**Fig 1:**
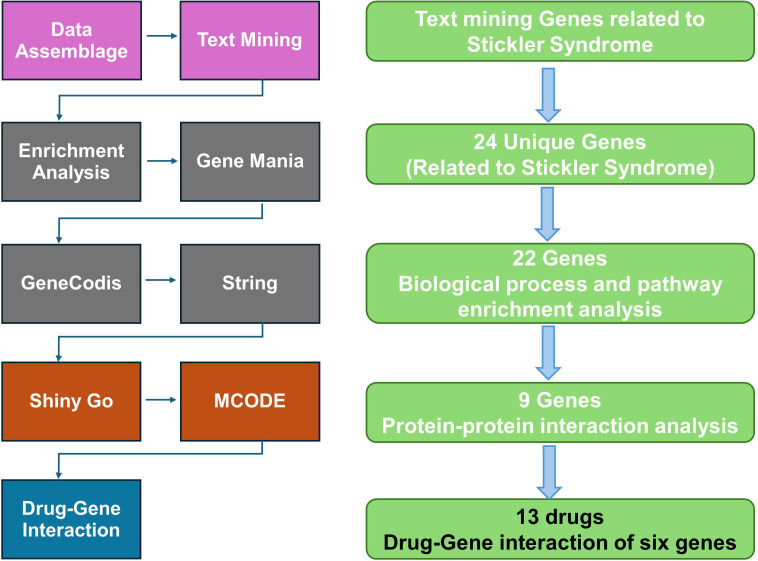
An overview of the methodological approach, including the flowchart and overall research design.

Using PubMed2Ensembl (http://pubmed2ensembl.ls.manchester.ac.uk), a text mining analysis was conducted to find genes linked to SS as described previously [13,14]. We used the search terms “Stickler syndrome,” “Hereditary Progressive Arthro-Ophthalmopathy,” and “Arthro-Ophthalmopathy” across 100,000 relevant document IDs to create a list of key genes associated with SS. The search parameters were deliberately narrowed to avoid overlaps with genes linked to other ocular, auditory, skeletal, and collagen disorders. The species dataset was limited to “Homo sapiens (GRCh37),” and the query results were filtered using “MEDLINE: PubMed ID.” Non-duplicated genes were extracted, and the text mining genes (TMGs) were identified as the intersection of gene hits from the three sets. As an additional validation step, the resulting gene list was manually cross-checked against established disease databases, including OMIM and DisGeNET, to confirm reported associations with Stickler syndrome or its core clinical features. Genes with evidence limited exclusively to other connective tissue disorders and without documented relevance to Stickler syndrome were not prioritized in downstream analyses. Fig 1, displays the research design overview and approach flowchart. All the databases’ contents were retrieved by January 2025.

### Analysis of enriched pathways and biological processes

The identified TMGs were analyzed for biological process annotations using the web-based tool GeneCodis. Using the TMGs as input, genes with significantly enriched biological processes associated to SS were chosen, and the genes implicated in the enrichment process were then investigated for functional links. GeneMania (version 3.5.3; https://apps.cytoscape.org/apps/genemania) was used to generate a gene-gene functional interaction network from the TMGs using the Cytoscape plugin version 3.10.3 and centrality measures were computed using the cytoHubba plugin. Adjusted p-values (FDR < 0.05), obtained using multiple-testing correction with the Benjamini–Hochberg procedure, were used as the basis for identifying significant GO and pathway enrichments and functional linkages. These standards guarantee that the observed enrichments are not the result of chance and are statistically significant. These requirements have been made clear in the Methods section.

### Protein-Protein Interaction (PPI) network construction and module evaluation

Based on GO analysis, we constructed a PPI network of 22 enriched genes using the STRING v11.5 database with the following parameters: Network type = evidence-based interactions (experimental, curated databases, and co-expression); Disconnected nodes = hidden to optimize network readability. As previously stated by Szklarczyk *et al* STRING includes over 3.1 billion interactions involving approximately 24.6 million proteins from 5,090 distinct species [15]. Furthermore, several key metrics from network theory were applied for analysis which are as following: Degree centrality (k) which indicates a node’s immediate importance in the network by counting the number of connections it has; Betweenness centrality (BC) which measures how often a node lies on the shortest path between other nodes, indicating its role in facilitating communication across the network; Closeness Centrality (CC) assesses how efficiently information can spread from a node to all other nodes, based on the average length of the shortest paths; Eigenvector centrality (EC), which indicates nodes connected to other highly influential nodes and gauges a node’s influence depending on the significance of its neighbors; Eccentricity indicates a node’s position in relation to the network’s periphery by measuring the greatest distance between it and any other node. It is believed that comprehending these metrics facilitates the analysis of network dynamics and structure, the identification of important nodes, and the evaluation of the network’s resilience and susceptibility. A confidence score of 0.900 was specified as the minimal criteria. Since the interactions in the PPI network are backed by substantial experimental and computational data, this high threshold guarantees that they are extremely dependable. The study’s foundation was thought to be the sub-network of these crucial proteins, which called for more research into the signaling pathways implicated in SS. By looking at the network architecture, the hub nodes were employed for centrality analysis after being grouped by a high score based on the network’s scale-free attribute. To find tightly coupled modules in the PPI network, we employed MCODE (Molecular Complex Detection, version 2.0.3), a built-in Cytoscape plugin with settings as: Degree cutoff = 2; node score cutoff = 0.2; K-score = 2; max depth = 100 [13,16]. High MCODE scores, which signify strongly interconnected nodes, were used to identify hub genes. Furthermore, nodes were regarded as hub genes if their degree centrality was higher than the 90th percentile.

### Identification of drug-gene associations

We used Drug-Gene Interaction Database (DGIdb, http://dgidb.genome.wustl.edu/) to ascertain existing drugs that potentially targets key hub genes we identified. DGIdb is well-known drug prediction database, which allows users to screen for drugs targeting specific genes of interest [17]. The DGIdb offers gene-drug interactions and potential druggability based on their gene category. The hub genes, previously identified through PPI network analysis based on centrality measures, were submitted using their official gene symbols. Within DGIdb, we applied filters to include only interactions supported by expert-curated sources such as DrugBank, TTD, and ChEMBL, and we prioritized clinically relevant compounds, particularly FDA-approved drugs. The retrieved drug-gene interaction pairs were reviewed to eliminate duplicates and low-confidence entries.

## Results

### Text mining-based screening revealed 24 genes related to Stickler Syndrome

**Fig 2:**
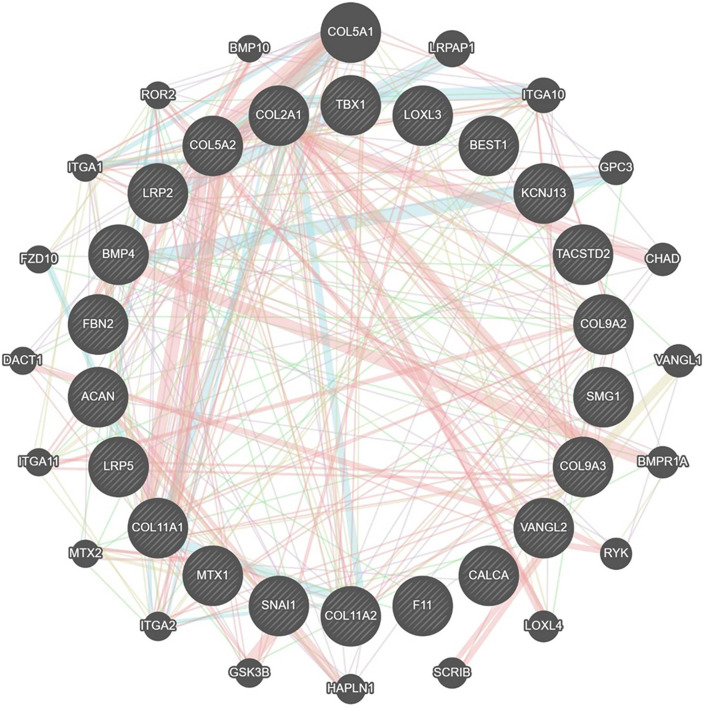
The graphical PPI network of all stickler syndrome related TMGs, created using Cytoscape as described in material and method section.

Using the TMG method, we identified 24 distinct genes linked to SS in humans. The network, co-expression analysis, genetic relationships, and pathways of the 24 TMGs evaluated by GeneMania are shown in Fig 2. Based on their GO and biological pathways, 22 of these genes were selected for enrichment analysis.

### Identified TMGs’ enrichment analysis revealed 10 KEGG pathways and 16 biological processes

Using GeneCodis, GO, biological process (BP), and KEGG, the most enriched terms that were directly associated with the pathology of SS were ascertained. Twenty-one genes were found to be significantly enriched by the GO and BP annotation analysis. The top 15 functions that were enriched were “Collagen Fibril Organization” (P = 4.80e-08), “Skeletal System Development” (P = 3.95e-06), “Ossification” (P = 3.36e-05), “Heart Morphogenesis” (P = 9.05e-05), “Sensory perception of sound” (P = 9.55e-05), “Negative Regulation Of Branching Involved In Ureteric Bud Morphogenesis” (P = 9.55e-05), “Roof Of Mouth Development” (P = 9.55e-05), “Cartilage Condensation” (P = 9.78e-05), “Cartilage Development” (P = 1.53e-04), “Outflow tract Septum Morphogenesis” (P = 2.24e-04), “Vasculature Development” (2.51e-04), “Limb Morphogenesis” (P = 2.55e-04), “Soft Palate Development” (P = 4.87e-04), “Skeletal System Morphogenesis “(P = 6.82e-04), “Extracellular Matrix Organization” (P = 3.44E + 10). A total of 10 major pathways involving 22 TMGs were identified through KEGG enrichment analysis. The five most significantly enriched pathways were “protein digestion and absorption” (P = 1.88e-08), “cytoskeleton in muscle cells” (P = 6.28e-05), “ECM-receptor interaction” (P = 7.31e-03), “Focal Adhesion” (P = 5.75e-02), “Human Papillomavirus Infection” (P = 1.72e-01), “PI3K-Akt Signaling Pathway” (P = 1.78e-01) involving 7, 6, 3, 3 and 3 text mining genes, respectively. Table 1 displays 15 enriched GO terms and Table 2 displays the KEGG analysis of 10 enriched molecular pathways of the TMGs.

**Table 1:** List of top 15 enriched GO biological process terms assigned to the text-mining genes.

Biological process	Gene In query Set	Total genes in Genome	P Value	Genes
Collagen Fibril Organization	6	64	4.80e-08	LOXL3, ACAN, COL11A2, COL11A1, COL5A2, COL2A1
Skeletal System Development	6	148	3.95e-06	ACAN, COL11A2, COL9A2, BMP4, COL5A2, COL2A1
Ossification	5	115	3.36e-05	BMP4, CALCA, COL11A1, COL5A2, COL2A1
Heart Morphogenesis	4	61	9.05e-05	TBX1, BMP4, COL11A1, COL2A1
Sensory perception of sound	5	165	9.55e-05	TBX1, COL11A2, COL11A1, LRP2, COL2A1
Negative Regulation Of Branching Involved In Ureteric Bud Morphogenesis	2	2	9.55e-05	BMP4, TACSTD2
Roof Of Mouth Development	4	71	9.55e-05	LOXL3, COL11A2, SNAI1, COL2A1
Cartilage Condensation	3	20	9.78e-05	ACAN, COL11A1, COL2A1
Cartilage Development	4	85	1.53e-04	COL11A2, BMP4, COL11A1, COL2A1
Outflow tract Septum Morphogenesis	3	28	2.24e-04	TBX1, BMP4, LRP2
Vasculature Development	3	30	2.51e-04	LRP5, BMP4, CALCA
Limb Morphogenesis	3	31	2.55e-04	LRP5, FBN2, COL2A1
Soft Palate Development	2	5	4.87e-04	TBX1, COL11A2
Skeletal System Morphogenesis	3	45	6.82e-04	COL11A2, COL11A1, COL2A1
Extracellular Matrix Organization	4	165	1.27e-03	COL9A2, COL11A1, COL9A3, COL5A2

**Table 2:** List of top 10 enriched KEGG pathways assigned to the text-mining genes.

Stickler syndrome KEGG pathway	Genes in the query set	Total genes in the genome	P- value	Genes
Protein Digestion and Absorption	7	103	1.88e-08	COL11A2, COL9A2, COL11A1, COL9A3, COL5A2, KCNJ13, COL2A1
Cytoskeleton In Muscle Cells	6	232	6.28e-05	COL11A2, FBN2, COL9A2, COL11A1, COL9A3, COL5A2
ECM-Receptor Interaction	3	89	7.31e-03	COL9A2, COL9A3, COL2A1
Focal Adhesion	3	203	5.75e-02	COL9A2, COL9A3, COL2A1
Human Papillomavirus Infection	3	332	1.72e-01	COL9A2, COL9A3, COL2A1
PI3K-Akt Signaling Pathway	3	361	1.78e-01	COL9A2, COL9A3, COL2A1
WNT Signaling Pathway	2	174	2.11e-01	LRP5, VANGL2
Fluid shear stress and atherosclerosis	1	141	3.39e-01	BMP4
Parathyroid hormone synthesis, secretion and action	1	115	3.39e-01	LRP5
Thyroid hormone signaling pathway	1	122	3.39e-01	BMP4

### PPI network construction, modular analysis, and key genes identification

**Fig 3:**
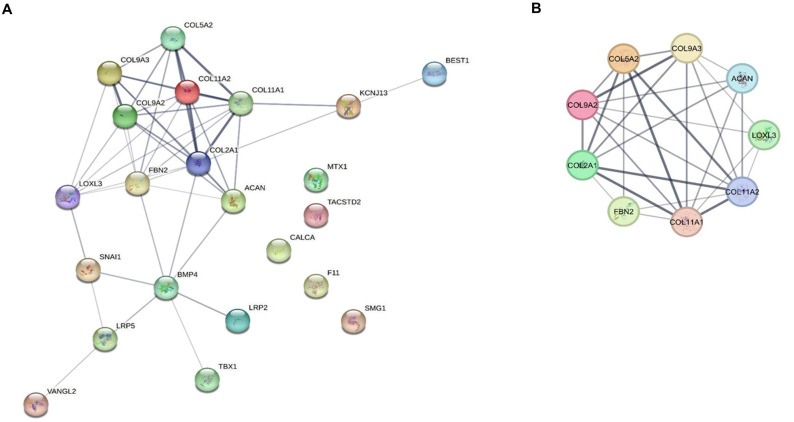
Identification and enrichment analysis of the TMGs. **(A)** PPI network of 22 Text mining genes visualized through Cytoscape; **(B)** Using MCODE, a single module of Hub Genes was obtained related to Stickler syndrome as visualized through Cytoscape.

**Fig 4:**
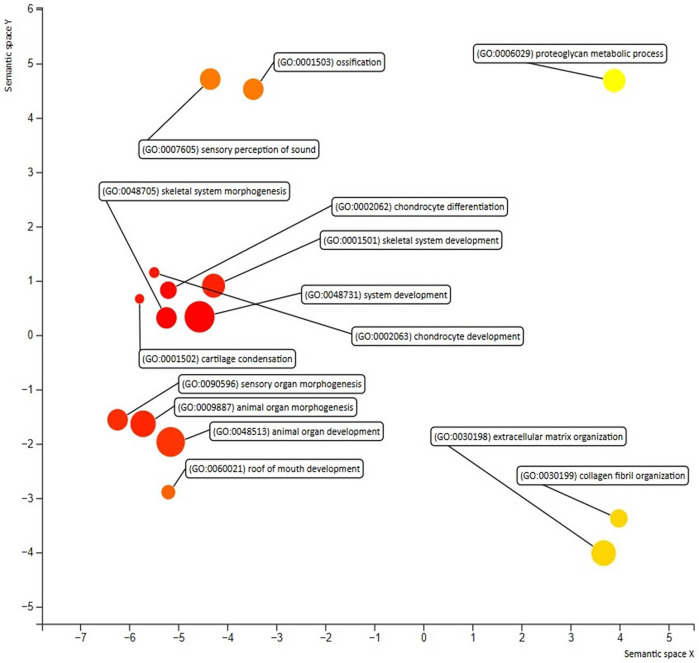
Illustration of the GO terms associated with the hub genes. Functional and pathway enrichment analyses were performed using the DAVID tool and visualized through the REVIGO web platform as described in material and method section.

As illustrated in Fig 3A, we used STRING to construct a PPI network with a low confidence score of less than 0.350 for each of the 22 text mining genes. There were 22 nodes and 46 edges in the network. We found a single important module by the modular analysis done with MCODE. COL2A1, COL9A2, COL9A3, COL5A2, COL11A1, COL11A2, ACAN, FBN2, and LOXL3 were the nine genes that made up this module (Fig 3B, Table 3). The REVIGO analysis of the hub genes, which was based on GO similarity, found several clusters that were mostly linked to the skeletal system development, collagen fibril organization, animal organ morphogenesis, animal organ development, ossification, extracellular matrix (ECM) organization, and proteoglycan metabolic process, as shown in Fig. 4.

**Table 3:** List of hub node genes in the PPI network which were identified with a filtering node degree ≥2.

Genes	Degree	MCODE cluster	MCODE SCORE
COL2A1	7	Clustered	4.7
COL9A2	7	Clustered	4.7
COL9A3	7	Clustered	4.7
COL5A2	6	Clustered	5.0
COL11A1	8	Clustered	4.7
COL11A2	8	Clustered	4.7
ACAN	5	Seed	5.0
FBN2	4	Clustered	4.0
LOXL3	4	Clustered	4.0

**Fig 5:**
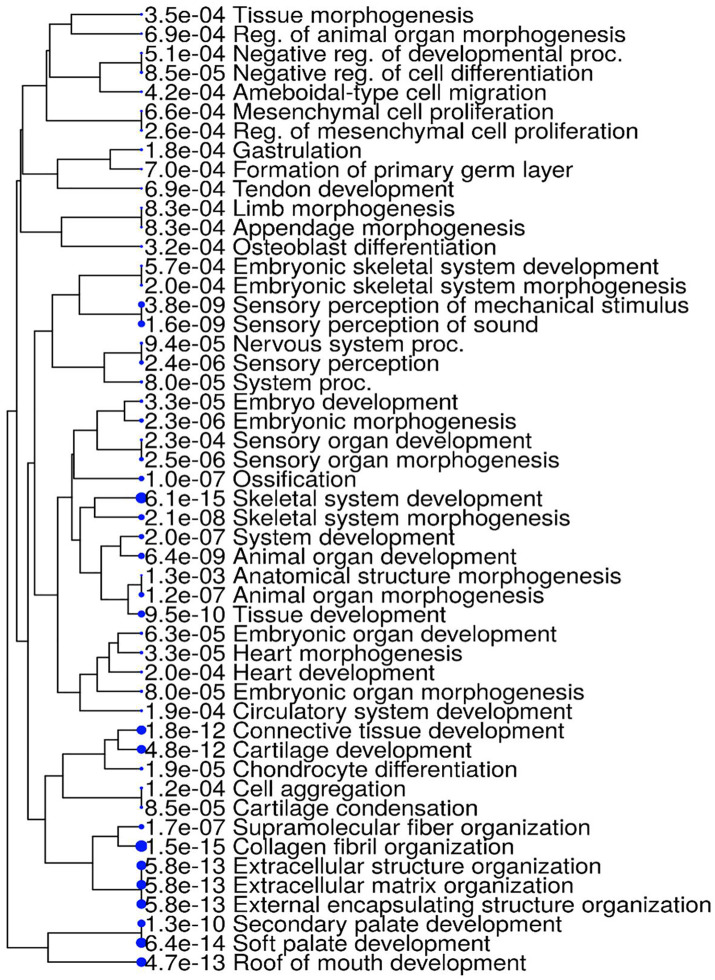
The illustration depicting the GO terms within the module identified as significant. A hierarchical tree was generated using the ShinyGO web server, revealing a strong correlation (P < 0.005). Functional and pathway enrichment analyses related to Stickler syndrome showed high enrichment scores.

To gain insights into the biological processes enriched among our hub genes, we performed GO enrichment analysis using KEGG and ShinyGo platform and visualized the results as a hierarchical clustering tree (Fig 5). The hierarchical tree highlights enriched terms associated with embryonic development, skeletal system formation, and connective tissue organization. Notably, highly significant GO terms included skeletal system development (p = 6.1e-15), collagen fibril organization (p = 1.5e-15), extracellular matrix organization (p = 5.8e-13), and roof of mouth development (p = 4.7e-13). Additional enriched categories included embryonic organ morphogenesis, heart development, cartilage development, and ossification, highlighting key processes in organogenesis and tissue differentiation (Fig 5). The clustering also highlighted sensory system-related processes, including sensory perception of sound (p = 1.6e-9) and sensory organ morphogenesis (p = 2.5e-6). Together, these results indicate that the analyzed gene set is strongly associated with developmental pathways, particularly those governing skeletal, connective tissue, and sensory system formation.

### Drug-gene interaction analysis of core genes

In our drug-gene interaction analysis, only six out of the nine identified hub genes were determined to be viable drug targets. As shown in Table 4, we found thirteen FDA-approved drugs predicted to exhibit significant interactions with these genes. Unfortunately, FBN2, ACAN, and LOXL3 did not exhibit any notable interactions, making them exceptions within this analysis. Most of the compounds are primarily used in diverse clinical contexts, including tissue remodeling, phosphodiesterase inhibition, anti-inflammatory action, and vasodilation. We believe that experimental validation and disease-specific functional assays in future are required to evaluate their efficacy, safety, and relevance in the context of SS.

**Table 4:** List of US FDA-approved drugs (with their present disease implication) that are predicted to target 5 of the 9 hub genes. * Indicates not currently US FDA approved for any use but holds Fast Track and Orphan Drug Designations. COPD means chronic obstructive pulmonary disease.

Gene	Drug (presently FDA-approved disease implication)	Regulatory Approval	Interaction	Interaction Score
COL11A2	OCRIPLASMIN (vitreomacular adhesion, retinal detachment)	Approved	Inhibitor	0.1373
	APREMILAST (Psoriatic arthritis, plaque psoriasis, Ulcers)	Approved	Inhibitor	0.5220
	IBUDILAST*	Fast Track	Inhibitor	0.3263
	COLLAGENASE CLOSTRIDIUM HISTOLYTICUM (Dupuytren’s contracture, Peyronie’s disease)	Approved	Inhibitor	0.1864
COL9A3	CILOSTAZOL (peripheral arterial disease)	Approved	Inhibitor	0.6215
	PAPAVERINE (vasospasm, visceral smooth muscle spasm)	Approved	Inhibitor	0.4834
	MILRINONE (severe congestive heart failure)	Approved	Inhibitor	1.450
	INAMRINONE (Short-term intravenous treatment of congestive heart failure)	Approved	Inhibitor	0.4579
COL2A1	COLLAGENASE CLOSTRIDIUM HISTOLYTICUM	Approved	Inhibitor	1.864
	OCRIPLASMIN	Approved	Inhibitor	0.4579
	ALBUTEROL SULFATE (reversible obstructive airway diseases)	Approved	Inhibitor	2.677
COL11A1	COLLAGENASE CLOSTRIDIUM HISTOLYTICUM	Approved	Inhibitor	0.1165
	CRISABOROLE (atopic dermatitis)	Approved	Inhibitor	0.4661
	ROFLUMILAST (severe COPD)	Approved	Inhibitor	0.5438
	OCRIPLASMIN	Approved	Inhibitor	0.0859
	IBUDILAST*	Fast Track	Inhibitor	0.2039
	APREMILAST	Approved	Inhibitor	0.3263
COL5A2	OCRIPLASMIN	Approved	Inhibitor	0.6869
	COLLAGENASE CLOSTRIDIUM HISTOLYTICUM	Approved	Inhibitor	0.9322
COL9A2	ENOXIMONE (acute heart failure)	Approved	Inhibitor	0.87
	CILOSTAZOL	Approved	Inhibitor	0.37
	ANAGRELIDE HYDROCHLORIDE (essential thrombocythemia)	Approved	Inhibitor	1.74
	INAMRINONE	Approved	Inhibitor	0.27
	MILRINONE	Approved	Inhibitor	0.87
	IBUDILAST*	Fast Track	Inhibitor	0.32
	PAPAVERINE	Approved	Inhibitor	0.29

## Discussion

As far as we know, there isn’t a therapeutic intervention available that can change the growing severity of stickler syndrome, a dominantly inherited connective tissue disorder that affects multiple systems, including the eyes, face, ears, and skeleton [4]. Since SS is an orphan disease known for limited patient groups, clinicians have not been successful to investigate the underlying mechanisms for this illness. We can speculate that using traditional variant discovery techniques such as NGS may be expensive, time consuming, and result in intricate data processing for variations that have not yet been discovered, including the assessment and analysis of biochemical pathways and genetic variants. Recently, we and several others have demonstrated that text mining is a useful method for generating disease hypotheses, as it can reveal previously unidentified links between genes and disease pathologies [13,16,18,19]. A combination of text mining, biological knowledge, and a bioinformatics methodology offer fresh perspectives on the possibilities of repurposing currently available drugs. SS is both genetically and phenotypically heterogeneous, with most cases involving mutations in genes related to collagen synthesis and others. Given this complexity, a comprehensive understanding of the molecular mechanisms underlying SS is crucial.

Our present study indicates that the genes linked to SS share similarities with several other signaling pathways, which may help find a diversified therapeutic targets and unique biomarkers. Our analysis revealed 22 significant TMGs that may be involved in the pathophysiology of SS, with enriched GO and BP terms associated with the critical functions such as collagen fibril organization, cartilage development, sensory perception of sound, tendon development, visual system development, ossification, eye morphogenesis, heart development, connective tissue development, and soft palate formation. Furthermore, the PPI network and enrichment analysis identified nine hub genes: COL2A1, COL5A2, COL9A2, COL9A3, COL11A1, COL11A2, ACAN, FBN2 and LOXL3. Type II collagen, the primary fibrillar collagen found in cartilage and the vitreous body, is encoded by COL2A1. Mutations in this gene result in vitreoretinal degeneration and skeletal abnormalities that are typical of SS [20]. Pathogenic variations in COL11A1 and COL11A2 are associated with autosomal-dominant and recessive types of SS that manifest as ophthalmic, auditory, and craniofacial abnormalities. These genes generate heterotrimeric fibrils with type II collagen [21]. Although COL5A2 is traditionally linked to Ehlers-Danlos spectrum illnesses, altered COL5A2 expression may be a factor in the connective-tissue fragility observed in SS [22]. COL5A2 encodes type V collagen, a regulator of fibril diameter and ECM stability. Early-onset retinal detachment has been linked to COL9A2 and COL9A3, which encode elements of type IX collagen and have been observed in recessive SS [10,23].

**Fig 6:**
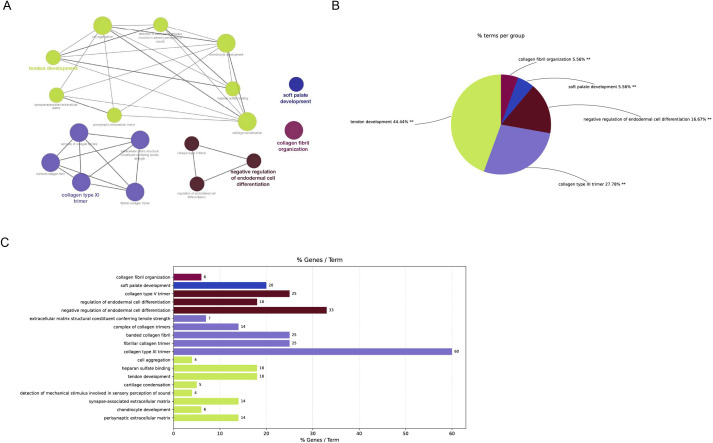
Functional characterization of the nine central hub genes within the module. **(A)** Key biological processes and pathways were visualized using the ClueGO plugin. **(B)** Functional and pathway distributions across the core genes are presented. **(C)** KEGG pathways and enriched GO terms are shown, with each pathway represented by a distinct color. A corrected P-value of less than 0.01 was considered statistically significant.

One of the main proteoglycans of the ECM of the growth plate and articular cartilage is aggrecan, which is encoded by ACAN gene. Aggrecan is essential for cartilage and bone morphogenesis and gives joints their hydrated gel structure, which is necessary for their load-bearing capabilities [24,25]. While ACAN is not established as a primary cause of SS, it is known to impact critical cellular processes that coincide with those impacted in the disorder. Previous studies have shown that patients with a wide range of short stature characteristics have been found to have at least 25 pathogenic ACAN mutations [26]. Similarly, another hub gene identified in our study, FBN2 encodes for Fibrillin-2, a crucial part of the ECM’s elastin fibers, which provide structural support and act as scaffolds for physiological functions. Pathogenic variants in FBN2 can disrupt microfibril structure or interfere with binding capacity, thereby weakening elastic fibers and impairing ECM-mediated signaling [27]. A few FBN2 mutations have also been identified in diseases other than SS [28]. So, it will be interesting to investigate for the mutational status of ACAN and FBN2 genes in SS patients. We also found LOXL3, a member of the lysyl oxidase (LOX) gene family. The first stage of collagen and elastin crosslinking, which is crucial for the biogenesis and structural integrity of connective tissues, is catalyzed by the prototypical LOX enzyme, an extracellular, copper-dependent amine oxidase. This function is likely conserved across the LOX family, as their amine oxidase activity depends on a highly conserved amino acid sequence at the C-terminal region. Many disorders, including malignant tumors, are caused by abnormal expression and activity of LOX family members [29]. There are few case reports in the literature which suggest LOXL3 to be mutated in SS patients [30–32]. Notably, in our analysis, the downstream genes LRP2 and GZF1 of LOXL3 did not appear as hub genes and were also not predicted to interact with any FDA-approved compounds. We believe that these insights have not only enhanced our understanding of disease mechanisms but also offer a foundation for exploring novel diagnostic markers and therapeutic targets. Together, these analyses indicate that the enriched gene set is functionally clustered around extracellular matrix organization and collagen trimer formation, which support processes central to tendon and cartilage development, craniofacial morphogenesis, and regulation of cell differentiation (Fig 6).

Our drug-gene interaction analysis identified thirteen FDA-approved compounds that may interact with these hub genes. We believe that these findings should be interpreted as hypothesis-generating observations rather than evidence of therapeutic efficacy. Among them, ocriplasmin and collagenase clostridium histolyticum seems to be optimistic choices, since both directly target ECM components. Ocriplasmin is a proteolytic enzyme, which is FDA approved for the treatment of vitreomacular adhesion (VMA) in adults. VMA is associated with vision symptoms and can sometimes be used in cases with small macular holes. Ocriplasmin’s mechanism of action involves targeting of tractional processes that are clinically relevant in SS-associated vitreoretinal pathology. Similarly, collagenase clostridium histolyticum enzymatically digests collagen types I and III and is FDA-approved for conditions involving fibrotic tissue, such as Dupuytren’s contracture and Peyronie’s disease [33,34]. Its ability to enzymatically cleave collagen is of conceptual interest in whether similar approaches could modify pathological collagen adhesions observed in SS-associated vitreoretinal abnormalities. Additionally, several phosphodiesterase (PDE) inhibitors were also identified, including apremilast, roflumilast, cilostazol, milrinone, inamrinone, enoximone, crisaborole, and anagrelide hydrochloride which are FDA-approved for implications listed in Table 4. Since these drugs modulate intracellular signaling by increasing cyclic nucleotide levels (cAMP/cGMP), leading to anti-inflammatory, vasodilatory, or cardiotonic effects they can be speculated to contribute towards cartilage degeneration, ocular pathology, and connective tissue homeostasis in SS pathology [35]. Apremilast and roflumilast are clinically used for inflammatory conditions like psoriasis and COPD, suggesting possible utility in modulating inflammatory aspects of ECM remodelling in SS disease pathology [36]. Meanwhile, cilostazol and milrinone, known for their vasodilatory and inotropic effects, could hypothetically influence vascular or musculoskeletal components affected in SS [36]. The suggested compounds represent potential directions for future research, particularly for experimental validation and drug-repositioning studies in SS-relevant models. Drug-repositioning is the process of identifying a new therapeutic application for an existing medication [37,38]. It is an emerging paradigm in drug development, particularly effective in addressing uncommon or orphan diseases, stratified patient populations, and urgent public health issues. The importance and possible risks of drug repurposing at the population level were highlighted more recently by COVID-19, as while current molecules are well-positioned to address urgent clinical needs, they are also vulnerable to unfounded speculations [39,40].

While these drug-gene associations offer a promising starting point, it is crucial to interpret them within the limitations of *in silico* prediction. Functional validation, disease-specific modeling, and safety profiling are essential next steps to assess the feasibility of repurposing these agents for SS. Nevertheless, our findings provide a valuable framework for identifying mechanistically relevant therapeutic candidates targeting ECM dysregulation and connective tissue pathobiology.

## Conclusion

Through our *in silico* study, we concluded that the hub genes COL2A1, COL5A2, COL9A2, COL9A3, COL11A1, COL11A2, ACAN, FBN2 and LOXL3 are involved in the development of SS. These genes are primarily associated with functions related to skeletal system development, system development, animal organ morphogenesis, animal organ development, ECM organization, roof of mouth development, soft palate development, collagen fibril organization, which may lead to muscular degeneration. Since SS is an orphan disease with little pathology knowledge and significant genetic heterogeneity, we think our research is of particular interest. Given the inherent limitations of computational and text-mining approaches, further functional validation using cellular systems, animal models, and clinical data is a must to confirm the biological and therapeutic significance of these findings. Nonetheless, our study provides a systematic framework for prioritizing genes and pathways and may serve as a useful resource for guiding future mechanistic and translational research in SS.
